# Focal nodular hyperplasia in a 14-year-old child: A case report

**DOI:** 10.22088/cjim.12.0.460

**Published:** 2021

**Authors:** Farzan Vahedifard, Masoud Mortezazadeh, Abbas Mofidi, Mehdi Kashani, Alireza Sharifi Rayeni

**Affiliations:** 1. Iran University of Medical Sciences, Tehran, Iran; 2 Sina Hospital, Tehran University of Medical Sciences, Tehran, Iran; 3 Faculty of Medicine, Iran university of Medical Sciences, Tehran, Iran; 4 Department of Gastroenterology, Sina Hospital, Tehran University of Medical Sciences, Tehran, Iran

**Keywords:** Focal nodular hyperplasia, Child, Benign liver tumors, Conservative management

## Abstract

**Background:**

Focal nodular hyperplasia (FNH) is a benign rare liver neoplasm in children and includes only 2% of all pediatric liver tumors. Here we reported the case of a 14-year-old girl with vague flank pain who was managed conservatively.

**Case Presentation:**

Our case is a 14-year-old child (female), with a 5 cm diameter lesion in the right lobe of the liver in CT scan, and histologic findings compatible with FNH. A solid mass lobulated contour, intense enhancement with a hypodense central area, possibly indicative of central scar, was seen. Despite her mild flank pain we did not insist on surgical resection and managed her conservatively. Her pain resolved 2 weeks later and an imaging follow-up with ultrasound 6 months later showed no increase in size or numbers.

**Conclusion:**

FNH is an uncommon mass lesion in children. Our patient had mild symptomatic severity, and several guidelines recommend surgical treatment in this condition, but our team performed conservative and medical treatment for her and got the desired result. Therefore, the combination of these factors raises the importance of introducing the case. According to FNH’s nature, stability, complications, and evaluation of pain are essential to avoid unnecessary surgeries.

Hepatic tumors may be either malignant or benign, Focal nodular hyperplasia (FNH) is a nonmalignant hepatic tumor which is rarely seen in childhood and compromises up to 2% of all pediatric liver tumors and predominantly found in females with the age spectrum between 20 to 50 (1). Most of the FNH tumors are asymptomatic and found incidentally in abdominal imaging; however, some patients have nonspecific signs and symptoms such as vague abdominal pain or mass related symptoms. FNH is most often solitary (80 percent) and usually less than 5 cm in diameter, and the diagnosis made by characteristic features of imaging studies and biopsy is less needed. It is complicated in only rare cases and mostly managed conservatively, and if the surgery is indicated, the outcome is excellent (2). We herein report a case of a 14-year-old girl with a large 5 cm diameter FNH mass with vague flank pain who was managed conservatively despite her mild abdominal pain. FNH is generally not common and has more prevalence in the age of 20 and 50. In this case, we detected FNH in a child. Also, our patient had mild symptomatic severity, and several guidelines recommend surgical treatment in this condition, but our team performed conservative and medical treatment for him and got the desired result. Therefore, the combination of these factors raises the importance of introducing the case.

## Case presentation

A 14 –year-old teenager with no past medical history referred to the gastroenterology clinic with a complaint of chronic vague right flank pain 2 months ago, the severity of pain was mild with frequent pain-free intervals. There were no significant changes in the severity of pain during these periods, she also describes that she had a low level of pain and could ignore it most of the time.

**Figure 1:**
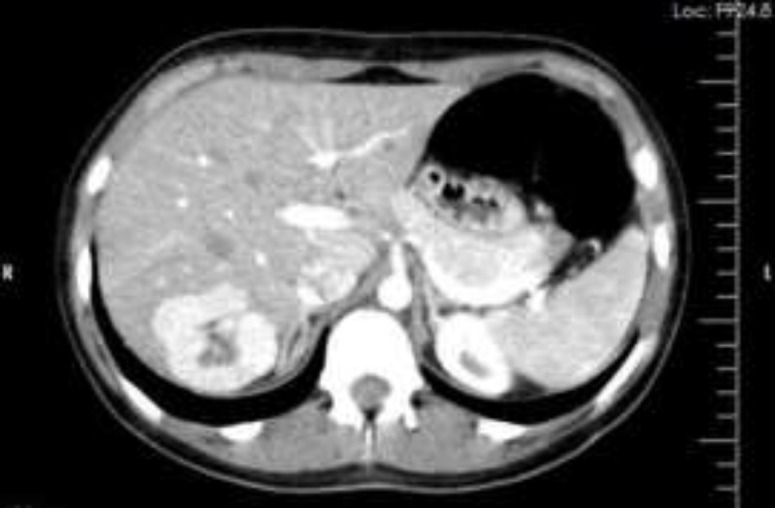
Enhanced axial CT scan through the liver in the arterial phase in a 14-year-old child. The mass demonstrates intense enhancement with a central scar. Solid mass lobulated contour, intense enhancement with a hypodense central area, which is possibly indicative of central scar

The physical exams were unremarkable and only positional right flank pain in lateral bending was noted. The lab tests were sent and CBC count, liver enzymes, alkaline phosphatase, and alpha-fetoprotein level all were within normal limits. The ultrasonography of the liver revealed an ill-defined 5 cm diameter hyperechoic homogeneous mass lesion in the right lobe. The CT scan demonstrates also 5 cm diameter mass with intense enhancement in the arterial phase in the right lobe in segments VII, VIII (figure 1).

**Figure 2:**
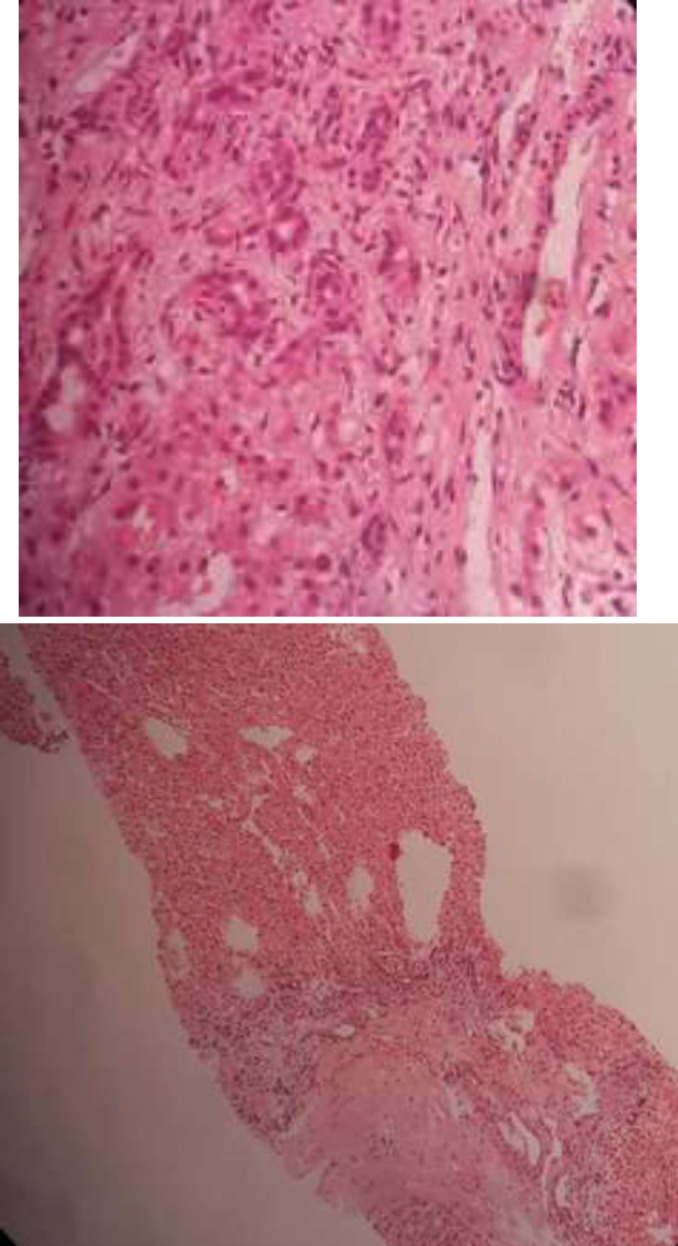
focal cirrhosis, fibrotic bands, prominent blood vessels, the proliferation of bile ductules which is compatible with focal nodular hyperplasia

The microscopic evaluation from core needle biopsy of the liver reported the liver tissue with distorted architecture, fibrotic bands, prominent blood vessels, the proliferation of bile ductules, and no evidence of viral effects or cellular atypia. All the histologic findings were compatible with focal nodular hyperplasia (figure 2).

According to the nature of FNH mass and definite diagnosis through biopsy, despite her mild flank pain we did not insist on surgical resection and managed her conservatively. Her pain resolved 2 weeks later and imaging follow-up with ultrasound 6 months later showed no increase in size or numbers.

## Discussion

FNH is a benign liver neoplasm, and it is described by hepatocyte hyperplasia and a central stellate scar, its pathogenesis is not well understood and maybe a vascular injury or environmental factors are indicative. Overall, the FNH is the second common benign tumor that is driven from the liver cell and is especially common in young women between the ages of 20 to 5, with a male/female ratio of 1:12 (3, 4). FNH is an extremely rare diagnosis in children and comprises up to 2 percent liver neoplasms in children (5), most patients’ clinical course is silent and found incidentally in abdominal imaging, however,some patients have nonspecific symptoms such as abdominal pain or mass related symptoms.

Abdominal pain is the most common presentation which has been described in symptomatic patients and usually the lesions are more than 4 cm by compressing adjacent organs or distention of Glisson’s capsule may cause abdominal pain but it is important to consider the severity of pain and quality of life, as we discuss in our case the pain was transient and surgery should be reserved for significantly symptomatic FNH masses and low threshold in pain evaluation could lead to unnecessary surgery (6).

Despite hepatic adenoma, complications such as tumor rupture, necrosis, and hemorrhage are also rare in FNH so conservative management in this lesion has favorable outcomes. Laboratory findings often do not demonstrate clinical significance, and tumor markers, such as AFP, are usually within normal limits and only 1/2 of cases have mildly elevated levels of gamma-glutamyl transpeptidase and in our reported case, all the mentioned laboratory results were within normal limits (7).

The majority of reports consider measurement of AFP and glutamine synthetase immunostaining for assessing other differential diagnoses such as hepatoblastoma in suspected patients. Imaging, ultrasound, and MRI have 82.6% sensitivity and 97.4% specificity for the FNH diagnosis and if the diagnosis remains uncertain, a liver biopsy is helpful (8). Ultrasonography (US) combined with duplex Doppler US is usually performed as first diagnostic imaging and if further confirmation is required, CT, MRI, angiography, and radionuclide imaging may be used.

US characteristics reported in our case were unable to distinguish the FNH from an adenoma or malignant lesions,optimal evaluation helical multi-phase CT scan with contrast during the hepatic arterial and portal venous phases was performed. A 5 cm hyperdense lesion during the hepatic arterial phase and hyperdense central scar during the delayed portal venous phase was reported (9). The management of asymptomatic patients is conservative and ultrasound examination every 6 months for three years is recommended and if no changes in size and numbers are reported these intervals could be longer (10).

Recent follow-up studies demonstrated that in 2/3 of the cases, the mass size remains stable while 1/4-1/3 of them reduced in size even complete remission is noted and only in few cases increasing in size and numbers was reported (11). In line with the studies of Bröker et al., 12 % of 162 confirmed FNH patients showed an increase in mass size over the 6 months and no adverse outcomes were detected in patients with growing FNHs. Therefore after a certain diagnosis, increasing in size without related symptoms is not a good reason for surgical resection and could be managed by close follow-up imaging studies (12).

Surgical resection should be considered if there is an evidence of progressive growth, indefinite diagnosis or hepatitis B virus carriage, or in large lesions, the exact size and cut off in children is not recommended and it seems that it depends on the age, body weight, and height, however in patients with persistent symptoms, it is wise to consider surgery (1, 13).

Embolization and vascular occlusion are also considered in symptomatic but un-resectable lesions (14). Since FNH is a benign lesion, it is generally described non-operatively in adults. However, most children with FNH undergo resection due to complications, expanded size, or failure to rule out malignancy (15). The results of our study suggest that according to the FNH characteristic features such as stability, rare complications, absence of malignant transformation and possibility of reduction in size; conservative management is preferred to surgery in mild symptomatic cases.

Ethics:In this study, no additional costs and procedures were imposed on the patient. Due to not mentioning the patient's details, our case report received the ethics code R.TUMS.SINAHOSPITAL.REC.1399.046 from the Ethics Committee of Tehran University of Medical Sciences.
